# Supplementary material for: A 3D printable alloy designed for extreme environments
Source: Nature. 2023 Apr 19;617(7961):513–8. doi: 10.1038/s41586-023-05893-0 (PMC10191839; doi:10.1038/s41586-023-05893-0)
Supplement: Supplementary file 1 — Supplementary Figs. 1–3 and Table 1. [file 41586_2023_5893_MOESM1_ESM.pdf]

nature portfolio

https://doi.org/10.1038/s41586-023-05893-0

Supplementary information

A 3D printable alloy designed for extreme environments

In the format provided by the authors and unedited

Supplementary Data:

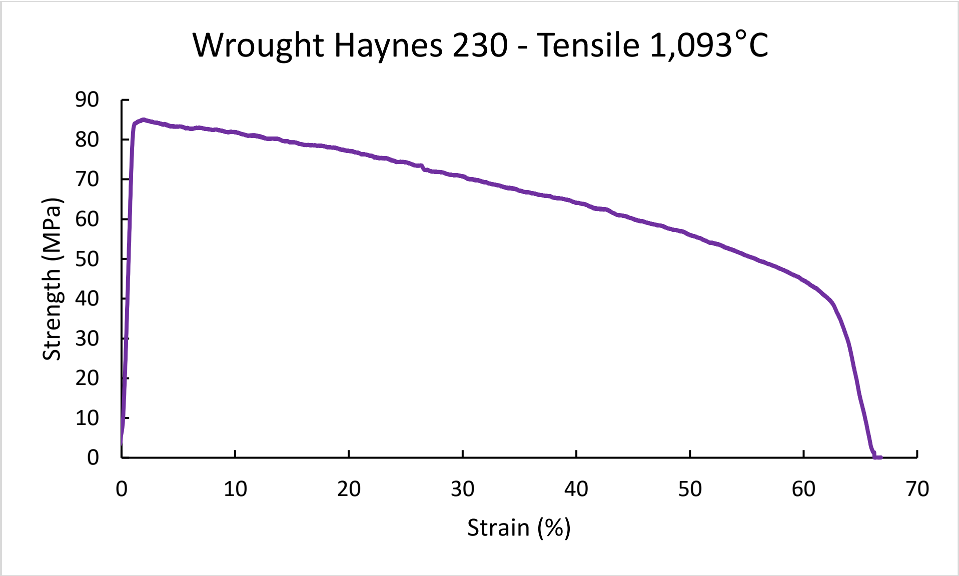

Supplementary Fig. 1: Elevated Temperature Wrought Haynes 230 Tensile Test. Tensile curve of wrought Haynes 230 at 1,093°C.

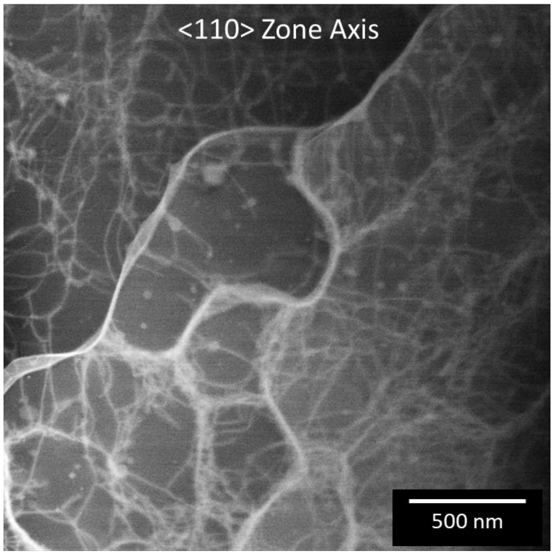

Supplementary Fig. 2: Creep deformation in GRX-810. A A BF-STEM DCI micrograph of dislocation sub structure from the 31 MPa GRX-810 creep sample that failed after 2500 h. The dislocation structure appears similar to that present in pre-tested GRX-810.

Supplementary Table 1: Alloy Name and Compositions. A table of each alloy and its corresponding composition that was referenced and described in the manuscript.

Alloy	Ni	Cr	Co	Mo	W	Nb	Ta	Al	Ti	Re	Fe	B	C	Y_2O_3
**Hastelloy X**	41.9	21	1	9	1	0	0	7	1	0	18	0	0.1	0
**Inconel 718**	52.56	19	1	3	0	5	0	0.5	0.9	0	18	0	0.04	0
**Inconel 617**	55.13	22	12.5	9	0	0	0	1	0.3	0	0	0	0.07	0
**Haynes 230**	61.6	22	0	2	14	0	0	0.3	0	0	0	0	0.1	0
**Haynes 188**	22	22	42	0	14	0	0	0	0	0	0	0	0	0
**Inconel 625**	62.95	21.5	0	9	0	3.6	0	0.2	0.2	0	2.5	0	0.05	0
**Haynes 233**	48	19	19	7.5	0.3	0	0.5	3.3	0.5	0	0	0.004	0.1	0
**NiCoCr-ODS**	33.7	30.7	34.7	0	0	0	0	0	0	0	0	0	0	1
**NiCoCr**	34	31	35	0	0	0	0	0	0	0	0	0	0	0
**ODS-ReB**	33.0	30.0	34.0	0	0	0	0	0	0	1.5	0	0.003	0	1
**GRX-810**	31.2	33	29	0	3	0.75	0	0.3	0.25	1.5	0	0	0.05	1
**GRX-810 non-ODS**	31.5	33.3	29.3	0	3	0.75	0	0.3	0.25	1.5	0	0	0.05	0

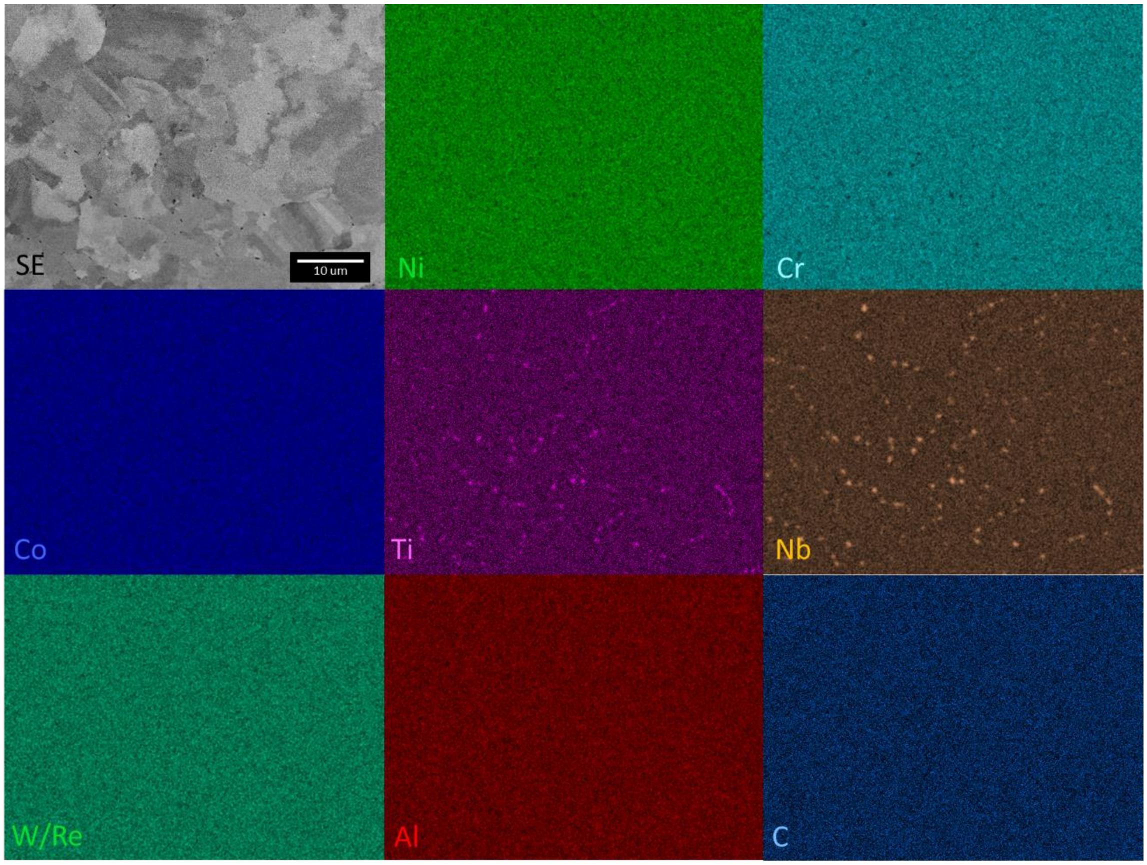

Supplementary Fig. 3: HIP GRX-810 Microstructural Characterization. SEM-EDS chemical maps of HIP GRX-810 revealing Nb and Ti rich MC carbides along grain boundaries. No other elemental segregation or phase formation is observed at this length scale.
